# An auxiliary diagnostic model based on joint learning of brain and lung data

**DOI:** 10.3389/fmed.2025.1593074

**Published:** 2025-10-03

**Authors:** Lishan Ye, Li Li, Fangfang Hua, Yi Yang

**Affiliations:** ^1^Department of Automation, Tsinghua University, Beijing, China; ^2^Department of Mechanical Engineering, Tsinghua University, Beijing, China; ^3^School of Communication Engineering, Hangzhou Dianzi University, Hangzhou, China; ^4^Zhejiang Hehu Technology Co. Ltd., Zhejiang, China; ^5^School of Reliability and Systems Engineering, Beihang University, Beijing, China; ^6^Peng Cheng Laboratory, Shenzhen, China; ^7^Hangzhou Innovation Institute, Beihang University, Zhejiang, China

**Keywords:** multi-disease, brain and lung data, classification, segmentation, datasets augmentation

## Abstract

Artificial intelligence has significantly improved diagnostic accuracy and efficiency in medical imaging-assisted diagnosis. However, existing systems often focus on a single disease, neglecting the pathological connections between diseases. To fully leverage multi-disease information, this paper proposes an auxiliary diagnostic model based on joint learning of brain and lung data (ADMBLD), aiming to enhance the comprehensiveness and accuracy of diagnoses through cross-disease correlation learning. The model integrates imaging data and clinical history of brain and lung diseases to identify potential correlations between different diseases. Experimental results show that the model trained on both brain and lung data outperforms those trained separately, validating the effectiveness of the multi-disease joint learning diagnostic model. This confirms that integrating multi-disease information captures latent pathological relationships, overcoming the limitations of single-disease models, thereby providing clinicians with more precise and comprehensive diagnostic support and demonstrating its potential in advancing intelligent diagnostic systems.

## 1 Introduction

Currently, artificial intelligence has made significant progress in medical imaging-assisted diagnosis, but many diseases are interconnected, especially across different organs. For example, lung (1–3) diseases like COPD are closely linked to cardiovascular conditions. Ignoring these cross-disease correlations can lead to incomplete diagnoses and affect treatment plans. Most current systems focus on single-organ imaging, like generating reports based solely on chest CT scans. In multi-disease diagnosis, combining medical reports with imaging data is essential for accurate diagnosis, utilizing techniques such as report segmentation and image classification to provide comprehensive support.

In medical report segmentation (4, 5), early research focused on overall classification of sentences or paragraphs, with medical text segmentation relying heavily on traditional machine learning methods. For instance, Chang et al. (6) used support vector machines for medical text classification, improving accuracy by incorporating medical dictionaries and rules. Li et al. (7) proposed a hierarchical Bayesian non-parametric model that successfully mined semantically coherent disease topics by integrating word distance information for semantic segmentation. In recent years, with the development of large language models, some studies have started using models like BERT for text semantic segmentation. Yang et al. introduced the BERT-BiGRU-CRF model, leveraging BERT to address ambiguity in text and enhance entity recognition accuracy. However, these models faced limited generalization performance in specific medical domains, leading to a decrease in accuracy. To improve large model performance in the medical field, Yao et al. (8) fine-tuned the BERT model, further enhancing its capabilities in medical text processing.

In medical image classification (9, 10), traditional methods primarily relied on manual feature extraction and classic machine learning algorithms. Researchers would construct classifiers through image preprocessing (e.g., denoising, enhancement) and feature extraction (e.g., texture features, shape features). However, these manual feature-based and classical machine-learning approaches often suffer from low classification accuracy and poor robustness. In recent years, neural network-based CT image classification methods have been introduced. For example, Hong et al. (11) developed a global reference framework by identifying head bounding boxes and sequentially locating other body parts. Zhang et al. (12) proposed a supervised method based on 3D CT image spatial information, improving the accuracy of body part recognition.

After report segmentation and image classification, the obtained multi-disease information can be modeled. In recent years, research based on methods such as Bayesian networks has emerged. For example, Heckerman et al. (13) designed the Pathfinder expert system, which uses a Bayesian network to diagnose over 60 diseases and 100 symptoms, significantly improving diagnostic accuracy. Constantinou et al. (14) developed a data-driven Bayesian network generation framework that uses the EM algorithm to learn conditional probabilities between variables. This framework can analyze four types of correlations: indirect causal effects, indirect evidential effects, common causes, and common effects. Despite progress in studying multi-disease interactions, particularly in using Bayesian networks and knowledge graphs (15) to improve diagnostic accuracy, challenges such as reliance on expert evaluations, subjectivity, and low computational efficiency still remain. Brain and lung diseases involve complex mechanisms and diverse clinical manifestations, and existing technologies face difficulties in data fusion and model construction. More efficient probabilistic acquisition methods and reasoning algorithms are needed. Therefore, we propose an auxiliary diagnostic model based on joint learning of brain and lung data. The innovations of our model are as follows:

- We employ data augmentation to perform preliminary feature extraction on the collected brain and lung data, thereby obtaining labeled reports and imaging data.
- We propose an auxiliary diagnostic model based on joint learning of brain and lung data. This model utilizes a small amount of manually labeled data, combined with brain and lung data, and employs methods such as smoothing, data augmentation, and transfer learning to significantly enhance the quality of medical report generation.

## 2 Method

The model in this study is divided into two parts. The first part involves dataset augmentation, where feature extraction is done through sequence classification and report segmentation, yielding annotated reports and imaging data. The second part focuses on model training, using a small portion of labeled data to train the sequence classification and report segmentation models, which are then applied to classify image sequences and segment report texts for the remaining data.

### 2.1 Datasets introduction

The brain dataset includes 22,429 samples from 10 hospitals, divided into a training set of 21,429 samples, a validation set of 400 samples, and a test set of 600 samples.The lung dataset contains 9,725 samples from 14 hospitals and health centers, split into 9,225 for training, 200 for validation, and 300 for testing.

Each examination dataset includes medical images and corresponding report data. The images mainly consist of CT scans of the brain and chest-lung regions. The report data contains clinical history, image description, and imaging diagnosis. Clinicians fill out the clinical history, while radiologists write the image description and diagnosis, which provide details of the image features, findings, disease diagnosis, and further examination recommendations.

### 2.2 Datasets augmentation

#### 2.2.1 Sequence classification

For imaging sequences, sequence classification aims to categorize them into brain, chest-lung, or other body regions. A straightforward approach to sequence classification is to make an initial determination based on the label information in the DICOM files that store the images. For instance, the Series Description field in DICOM often provides clues regarding the body region, to some extent. However, due to variations in storage practices across different hospitals and devices, the contents of labels such as Series Description can vary greatly and are often inconsistent. Relying solely on this information for classification is frequently problematic. To address this issue, we employed manual annotation to label a subset of the imaging sequences. Specifically, for the constructed brain and lung datasets, we randomly selected 1,873 imaging sequences for manual annotation, classifying them into categories such as brain, chest, and other regions. The labeled data were then split into training and testing sets at an 80:20 ratio.

#### 2.2.2 Report segmentation

For a case report with imaging data from multiple (16, 17) body regions, we segment the “image description” section to extract descriptions for specific regions like the brain and lungs. The output is a sequence of the same length, where each position corresponds to a body region. This task, known as report segmentation, aids in subsequent experiments.

**Figure 1:**
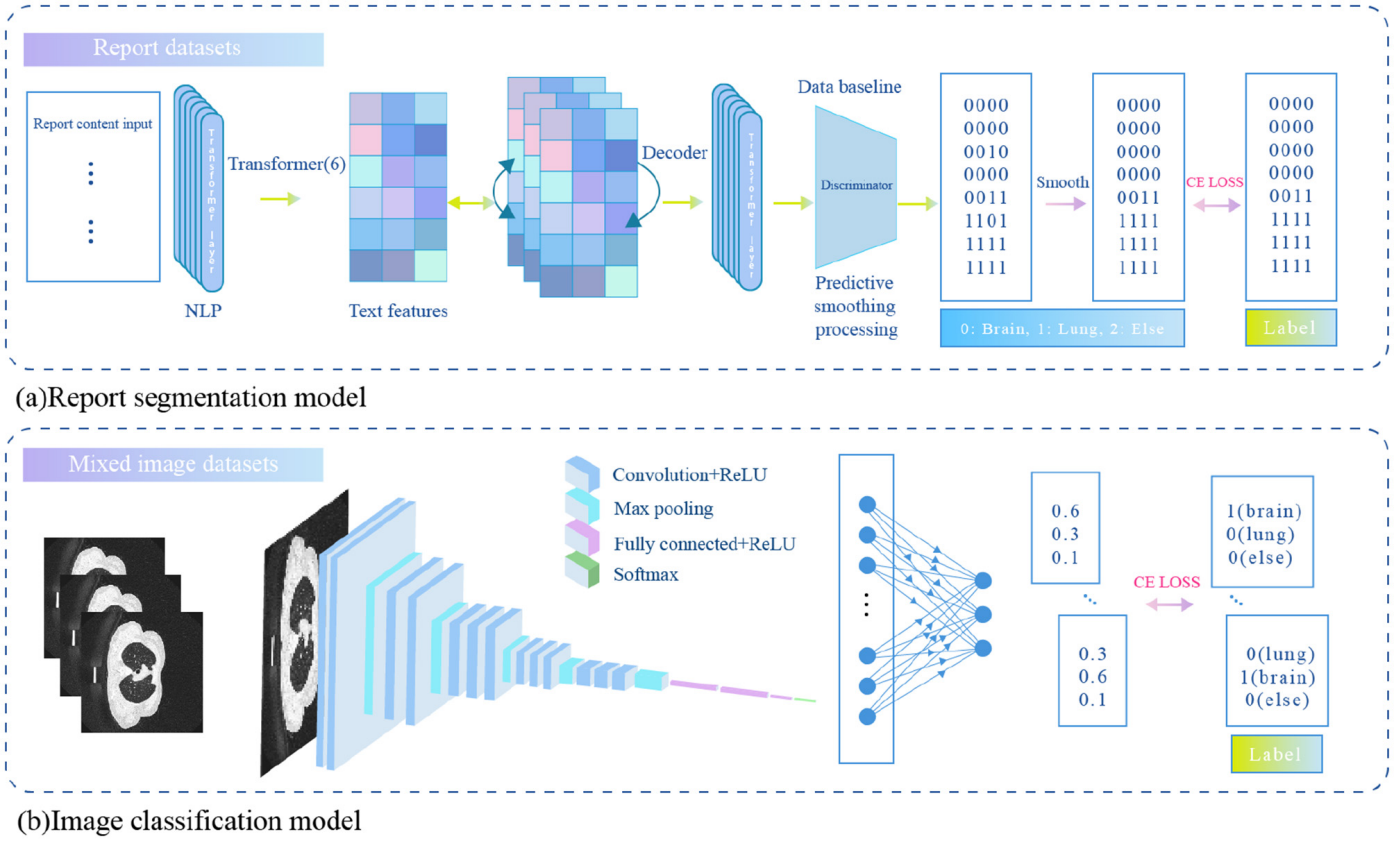
An auxiliary diagnostic model based on joint learning of brain and lung data.

To automatically perform report segmentation on large-scale reports and reduce the need for manual annotation, we trained a text processing model capable of automatically segmenting the imaging description text. Specifically, we extracted 1,100 reports from the brain and lung datasets, and manually annotated the different body regions described in the reports. The annotation labels included “brain,” “lung,” and “other.” To minimize labeling costs, we manually annotated only a small portion of the dataset. Based on this manually annotated data, we trained a sequence classification model and a report segmentation model to classify image sequences and segment report texts for the remaining data. The overall architecture of the model is shown in Figure 1. The primary mathematical symbols used in our model are detailed in Table 1.

**Table 1:** Notation and description.

**Notation**	**Description**
$X_{v}^{i}$	Medical image sequences
${ŷ}_{v}^{i}$	Predicted categories of medical imaging sequences
$y_{v}^{i}$	True categories of medical imaging sequences
$x_{t}^{i}$	Image description
${ŷ}_{t}^{i}$	Segmentation of predicted image descriptions
$y_{t}^{i}$	Segmentation of true image descriptions
*D*_*t*_	Text generation model
*E*_*v*_	Image encoding model
*y*_*t*_	Target text sequence

#### 2.2.3 Sequence classification model

The sequence classification model is designed to automatically classify the body region to which a medical image belongs based on an input sequence of images. Let the sequence be denoted as $x_{v}^{i}=\left[x_{v}^{i1},x_{v}^{i2},\ldots ,x_{v}^{iM}\right]$, where the sequence contains **M** slices of images, and the label for the sequence is $y_{v}^{i}$, representing the body region category. The sequence classification model is composed of a ResNet18 model as the image feature extractor and a fully connected layer (*f*_*c*_) as the classifier. Assume the input is a single slice RGB image $x_{v}^{ij}\in R^{k\times h\times 3}$ with 3 channels. The sequence classification model outputs a predicted probability ${ŷ}_{v}^{ij}\in R^{3}$, representing the probability that the slice belongs to each of the three categories. The specific calculation formula is as follows:


(1) $${\hat{y}}_{v}^{ij}=fc\left(ResNet(x_{v}^{ij}\right)$$

Since an image consists of multiple slices, we calculate the category prediction for the sequence by averaging the predicted results of a subset of slices. The specific formula is as follows:


(2) $$\begin{array}{l} {ŷ}_{v}^{i}=\frac{1}{M}\overset{M}{\underset{j=1}{\sum }}{ŷ}_{v}^{ij} \end{array}$$

The model parameters are ultimately trained using the cross-entropy loss function. The specific loss function is as follows:


(3) $$\begin{array}{l} L=-\overset{N}{\underset{i=1}{\sum }}y_{v}^{i}\cdot \log\left({ŷ}_{v}^{i}\right) \end{array}$$

where **N** denotes the number of labeled data points.

#### 2.2.4 Report segmentation model

The report segmentation model is capable of automatically segmenting the body parts corresponding to each sentence in the input image description. Let the input image description be denoted as $x_{t}^{i}=\left[x_{t}^{i1},x_{t}^{i2},\ldots ,x_{t}^{iM}\right]$, where **M** is the number of words in the description. The report segmentation label is represented by a corresponding encoding of the same length, denoted as $y_{t}^{i}\in R^{M}$, where each element of the label corresponds to the body part described by the word at the respective position. Since the label categories include “brain,” “chest and lungs,” and “others,” the encoding is defined as follows: the brain is labeled as 1, chest and lungs as 2, and others as 3.

The report segmentation model is a Transformer model consisting of 6 encoder layers. The maximum token length (sequence length) for the input sequence is 500. First, the input image description $x_{t}^{i}$ is tokenized to obtain its corresponding textual representation $f_{t}^{i}=\left[f_{t}^{i1},f_{t}^{i2},...,f_{t}^{iM}\right]\in R^{M\times d}$, where *d* represents the dimensionality of the text representation. This representation is then augmented with position encoding and fed into the Transformer encoder, resulting in the textual representation of the next layer. The specific calculation formula is as follows:


(4) $$\begin{array}{l} f_{t}^{i\left(l+1\right)}=E_{t}\left(f_{t}^{i\left(l\right)}\right) \end{array}$$

where *E*_*t*_ denotes a single layer of the Transformer encoder, $f_{t}^{i\left(l\right)}$ represents the textual representation at the *lth* layer, and $f_{t}^{i\left(0\right)}=f_{t}^{i}$.

After the 6 layers of encoding, the final representation $f_{t}^{i\left(6\right)}$ is obtained and subsequently passed through a fully connected layer for body part classification. The specific calculation formula is as follows:


(5) $$\begin{array}{l} {ŷ}_{t}^{i}=softmax\left(fc\left(f_{t}^{i\left(6\right)}\right)\right) \end{array}$$

Where ${ŷ}_{t}^{i}\in R^{M\times 3}$ represents the predicted body part category. The model parameters are ultimately trained using the cross-entropy loss function. The specific loss function is as follows:


(6) $$\begin{array}{l} L=-\overset{N}{\underset{i=1}{\sum }}y_{t}^{i}\cdot \log\left({ŷ}_{t}^{i}\right) \end{array}$$

where **N** denotes the number of labeled data points.

In order to improve the performance of the report segmentation model, we further employ the following optimization methods.

##### 2.2.4.1 Prediction smoothing

**Figure 2:**
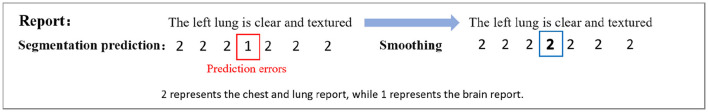
Predictive smoothing.

Considering that descriptions of the same body part in medical reports are usually continuous, a smoothing strategy is proposed. When the predicted category at a given position differs from that of the adjacent position, and the adjacent categories are the same, the predicted category will be adjusted to match the adjacent category. This approach enhances the model's ability to capture continuous descriptions, as shown in Figure 2.

##### 2.2.4.2 Data augmentation

To enhance the model's generalization, a text data augmentation method based on random editing is proposed. During training, each position has a 2% chance of randomly inserting or deleting characters, with the label sequence adjusted accordingly. Additionally, words are randomly replaced with synonyms, sentence order is shuffled, and minor grammatical or spelling errors are introduced to increase diversity. This approach expands the training data distribution and reduces overfitting.

##### 2.2.4.3 Transfer learning

To leverage the semantic information in large-scale unlabeled report texts, a transfer learning strategy is used for pretraining and fine-tuning the segmentation model. The text processing model is initially trained on a large corpus of unlabeled reports to learn general textual features. Then, the trained weights are transferred to the report segmentation model and fine-tuned on labeled data to better adapt to the specific needs of the segmentation task.

##### 2.2.4.4 Multi-task learning

The report segmentation task is jointly trained with related tasks, which enhances the model's understanding of medical texts. This approach enables the model to share lower-level text features and optimize across multiple tasks, improving overall performance.

### 2.2.5 Medical report generation

Assume the input report prefix is $f_{t}=\left[f_{t}^{1},f_{t}^{2},...,f_{t}^{n}\right]\in R^{n\times d}$, and the input medical image is $x_{v}\in R^{k\times h\times D}$, where *k, h*, and *D* represent the length, width, and number of channels of the input image, respectively. The image encoder *E*_*v*_ takes the medical image *x*_*i*_ as input and outputs the image features $f_{v}\in R^{D\times d}$, where *d* denotes the dimensionality of the image features. Subsequently, the image features *f*_*v*_ and the text representation *f*_*t*_ are jointly input into the text generation model to obtain the input for the next layer.

After *L* layers of encoding, the output sequence representation is passed through a linear mapping layer followed by a softmax layer, resulting in the prediction of the next word in the current text, denoted as ${ŷ}_{t}^{\left(v\right)}$. To supervise the model's performance in the report generation task, the cross-entropy loss is used to constrain the model's output. The objective is to maximize the similarity between the output sequence and the target sequence. The specific loss function is as follows:


(7) $$\begin{array}{l} L=-\overset{T}{\underset{t=1}{\sum }}\overset{V}{\underset{v=1}{\sum }}y_{t}^{\left(v\right)}\log\left({ŷ}_{t}^{\left(v\right)}\right) \end{array}$$

Where *T* is the length of the output sequence, *V* is the size of the vocabulary, $y_{t}^{\left(v\right)}$ is the true distribution of the target sequence at time step *t*, represented as a one-hot encoding, and ${ŷ}_{t}^{\left(v\right)}$ is the probability distribution generated by the model at time step *t*.

## 3 Experiments

To further investigate the brain-lung correlation and conduct cross-disease joint experiments, the brain and lung datasets were processed further. The enhanced datasets are shown in Table 2.

**Table 2:** Enhance healthcare datasets.

	**Sample set**			
**Datasets**	**Training set**	**Validation set**	**Test set**	**SUM**
Brain dataset	6,059	337	337	6,733
Lung dataset	5,091	283	283	5,657
Total	11,150	620	620	12,390

For the medical images in the brain and chest-lung datasets, we adopted random cropping and random sampling strategies to standardize the slices across different sequences. For the report texts, we set a maximum length to ensure consistent batch processing.

### 3.1 Experimental settings

We conducted experiments on an image-text multimodal model using a ResNet-18 image encoder built with the Pytorch framework. The experiments ran on an Nvidia Tesla T4 GPU (16GB memory) with a batch size of 8 for 50 epochs, at a learning rate of 3e-5. The results show that 50 epochs were enough for the model to converge on the training set and perform well on the validation set.

In this chapter, IOU (Intersection over Union), Precision, and Recall are used to evaluate the performance of the report segmentation model. **BLEU**, **ROUGE**_*L*_ and **CIDEr-D** are used to evaluate the performance of the medical image report generation task.

### 3.2 Experimental results and analysis

To evaluate the results of ADMBLD, we compare the segmentation accuracy when different modules are added. The performance of each model is shown in Table 3, with the optimal results highlighted in bold. Based on the results, the following conclusions can be drawn:

1. The baseline model achieved an IOU of 0.952, precision of 0.973, and recall of 0.979 on brain data, while for lung data, the three metrics were 0.890, 0.919, and 0.968, respectively. This suggests that brain data segmentation is relatively easier, likely due to the more regular structure of the brain, lower text complexity, and more concentrated image features.
2. After incorporating prediction smoothing, the segmentation performance for both brain and lung data improved, particularly the IOU, which increased to 0.958 and 0.904, respectively. This indicates that adjusting the continuity of model predictions effectively reduces fragmentation in category predictions, enhancing segmentation stability and consistency.
3. Further introducing data augmentation strategies led to an additional performance boost. Notably, for lung data, the IOU improved from 0.904 to 0.928, and precision significantly increased from 0.925 to 0.946. This demonstrates that diversifying training data helps enhance the model's generalization ability, especially in complex lung segmentation tasks.
4. Combining transfer learning resulted in optimal performance across both datasets. For brain data, the IOU increased to 0.962, with precision and recall reaching 0.977 and 0.985, respectively; for lung data, the IOU rose to 0.929, with precision and recall at 0.949 and 0.967, respectively. This shows that leveraging pre-trained model knowledge significantly improves the initial model parameters, enhancing overall segmentation performance.

**Table 3:** The segmentation result of report.

	**Brain dataset**			**Lung dataset**		
**Models**	**IOU**	**Precision**	**Recall**	**IOU**	**Precision**	**Recall**
Benchmark model	0.952	0.973	0.979	0.890	0.919	0.968
+ Smoothing	0.958	0.976	0.981	0.904	0.925	**0.978**
+ Data augmentation	0.957	0.973	0.984	0.928	0.946	0.975
+ Transfer learning	**0.962**	**0.977**	**0.985**	**0.929**	**0.949**	0.967

Bold values represent the best values in this set of data.

To investigate the correlation between different diseases and the impact of joint training on report generation performance, three experimental setups were designed in this section. These setups include training on the brain dataset alone, training on the lung dataset alone, and joint training on both the brain and lung datasets. Evaluations were conducted on both the brain and lung data.

The experimental results, shown in Tables 4, 5, lead to the following conclusions:

1. As shown in Table 4, for BLEU-1 to BLEU-4, the model trained solely on the brain dataset outperforms the one trained on the lung dataset, with the joint training model achieving the best performance. For the CIDEr-D metric, the joint training model also leads, with a score of 1.580. This suggests that joint training improves the model's generation ability for brain data, allowing it to better capture related semantics and expressions, leading to more accurate and coherent reports.
2. As shown in Table 5, the joint training model leads with a BLEU-1 score of 0.626. It also demonstrates strong performance across BLEU-2 to BLEU-4 metrics. For the ROUGE-L metric and CIDEr-D metric, the joint training model likewise shows advantages, scoring 0.532 and 0.409, higher than the models trained on brain or lung data alone. This indicates that joint training also has a positive impact on report generation related to lung data, improving the quality and diversity of reports generated for lung data.
3. Overall, while models trained on individual datasets for different diseases show some performance on their respective test sets, joint training demonstrates advantages across multiple evaluation metrics. This suggests that there is some correlation between datasets for different diseases, and joint training allows the model to learn richer features and knowledge. As a result, it enhances performance in report generation tasks for both disease-related datasets, offering a more optimal training strategy for future applications in medical report generation.

**Table 4:** When the model uses different training data, the report is generated on the brain dataset.

**Evaluate metrics**					
**Models**	**BLEU-1**	**BLEU-2**	**BLEU-3**	**BLEU-4**	**ROUGE-L**	**CIDEr-D**
Brain datasets	0.611	0.567	0.531	0.503	0.666	1.527
Lung datasets	0.599	0.554	0.519	0.491	0.659	1.465
Brain + Lung datasets	**0.632**	**0.582**	**0.543**	**0.513**	**0.667**	**1.580**

Bold values represent the best values in this set of data.

**Table 5:** The effect of the report generation on the lung dataset when the model uses different training data.

	**Evaluate metrics**					
**Models**	**BLEU-1**	**BLEU-2**	**BLEU-3**	**BLEU-4**	**ROUGE-L**	**CIDEr-D**
Brain datasets	0.549	0.478	0.428	0.390	0.507	0.295
Lung datasets	0.594	0.517	0.461	0.418	0.524	0.323
Brain + Lung datasets	**0.626**	**0.544**	**0.485**	**0.441**	**0.532**	**0.409**

Bold values represent the best values in this set of data.

## 4 Conclusion

To address the common limitation of existing intelligent diagnostic systems, which are often confined to single disease analysis, this paper proposes a multi-disease joint learning-based report generation model. By integrating brain and chest-lung medical imaging data with report text, the model explores potential correlations between diseases, thereby enhancing the comprehensiveness and accuracy of diagnoses. Experimental results show that multi-disease joint training can effectively uncover latent feature correlations between different diseases, significantly improving the performance of report generation for individual diseases. This validates the pathological correlations between different body parts and provides practical evidence for the further development of multi-disease intelligent diagnostic systems.

## Data Availability

The datasets presented in this article are not readily available due to patient privacy. Requests to access these datasets should be directed to yels21@mails.tsinghua.edu.cn.
